# Supplementary material for: Compression and Stretching of Confined Linear and Ring Polymers by Applying Force
Source: Polymers (Basel). 2021 Nov 30;13(23):4193. doi: 10.3390/polym13234193 (PMC8659573; doi:10.3390/polym13234193)
Supplement: Supplementary file 1 [file polymers-13-04193-s001.zip › polymers-1410188-supplementary.pdf]

Compression and stretching of confined linear and ring polymers by applying force

Wenduo Chen[1,2*], Xiangxin Kong[3], Wei Qianqian[1,2], Huaiyu Chen[1,2], Jiayin Liu[1,2] and Dazhi

1 School of Materials, Sun Yat-sen University, No. 135, Xingang Xi Road, Guangzhou, PR China, 510275
2 Shenzhen Campus of Sun Yat-sen University, No. 66, Gongchang Road, Guangming District, Shenzhen, P.R. China, 518107
3 Changchun Institute of Applied Chemistry, Chinese Academy of Sciences, Changchun, 130022, PR China
* Correspondence: chenwd29@mail.sysu.edu.cn

Supporting Information

In the compression process, a pair of external force is added on two ends/sides of a chain to stretch the polymer chain. Each simulation performs 10 000 000 steps to reach the steady states. Under a pair of compression force applied on the two ends/sides, the polymer chain is firstly compressed from the ends until it reaches the minimum size along the direction of external force. After that, the polymer chain is stretched from the condensed state, as shown the Fig.S1, Fig.S2, Fig.S3, and Fig.S4 in Supporting Information. We choose the chain states with the minimum size along the direction of external force as the finally collapsed states.

Figure S1 shows the trajectory of an individual chain in compression process for flexible linear and ring polymers in microchannel confinement under the strong force (*f*=1.0) and weak force (*f*=0.001). In Figure S1(a), when a couple of compression force *f*=1.0 are applied on the two ends, the linear polymer starts to shrink from the ends until it reaches the minimum size along the direction of external force. After that, the polymer chain is stretched from the condensed state under the constant force. Finally, it is full stretched to nearly the contour length under the synergistic effect of confinement and stretching. Similar to the flexible linear polymer, the flexible ring is also compressed from their two sides and then stretched under the constantly external force, as shown in Figure S1(b).

Figure S1(c) shows the linear polymers deformations are in the compression process and the following stretching process under the weak force (*f*=0.01). The ring polymers only present a less obvious compression under the same weak force (*f*=0.01), as shown in Figure S1(d). Because the two sides of ring polymers cannot be folded back, due to the strong excluded volume between the two strings of the ring polymer.

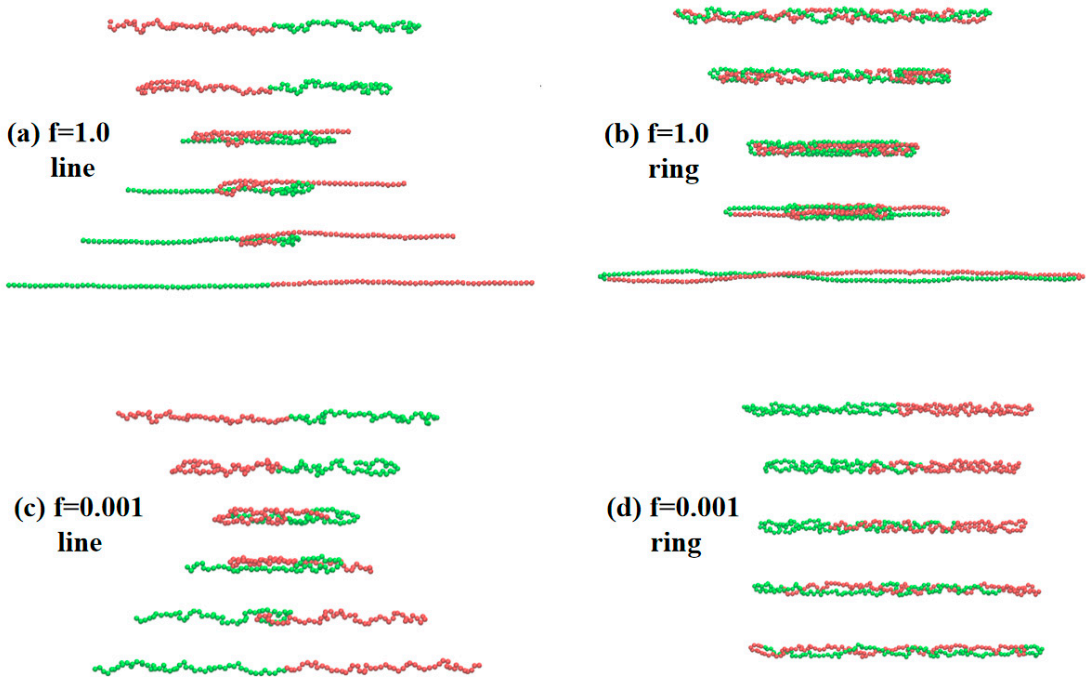

Figure S1. Compression processes of flexible linear and ring polymers in microchannel confinement (*d*=5) under the strong force (*f*=1.0) and weak force (*f*=0.001).

Under the constant force, the confined semiflexible chain with *Lp*=5 shrinks to the shortest length with three folded conformations, as shown in Figure S2. Then the chain is stretched from the collapsed states with the straight ends. The deformations and dynamics of semiflexible ring are agreement with the linear polymers in qualitative.

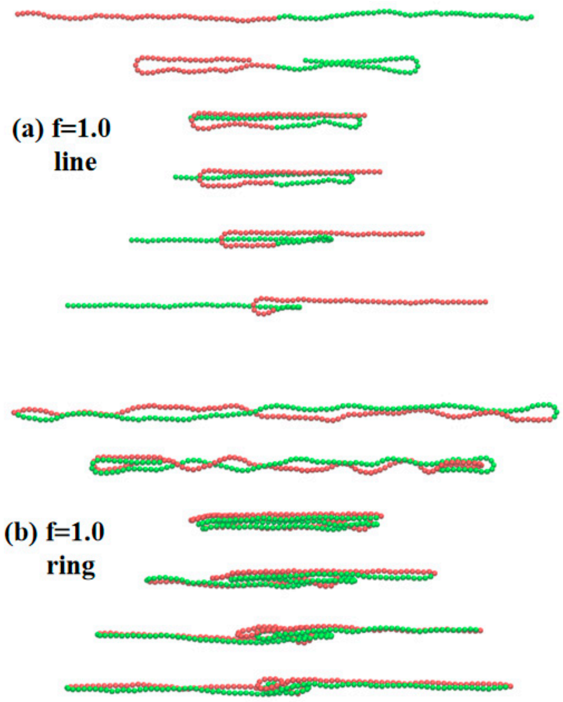

Figure S2. Compression processes of semiflexible linear and ring polymers (*Lp*=5.0) in microchannel confinement (*d*=5) under the external force *f*=1.0.

Figure S3 shows the chain length along the tube directions Lx and the thickness δ for different chain rigidities. The length Lx is the longest dimension of a polymer chain in the direction of channel axis, defined as $Lx = \max(x_i)-\min(x_j)$, where x_i and x_j is the x-coordinate for particles i and j. The chain thickness δ is defined as $\sqrt{(Gyy + Gzz)/2}$, where Gyy and Gzz are the squared radii of the gyration in the y and z directions, respectively.

Under the external force f=1.0, the flexible polymer chains show the compressional response, and then the stretching, independent on the topology. At the meantime, the thickness δ increases and show rapid decreasing afterwards (see Figure S3(a) and Figure S3(c)). The flexible linear chain is compressed quickly and then stretched from the condensed states. For the semiflexible chains, the compression processes become slowly due to the bending energy. However, the rigid chain shows only a little compression for the strong bending energy. The ring polymer show the similar deformations with the linear polymers, as shown in Figure S3(b) and Figure S3(d).

Here, we choose the chain states with the minimum size along the direction of external force as the finally collapsed states.

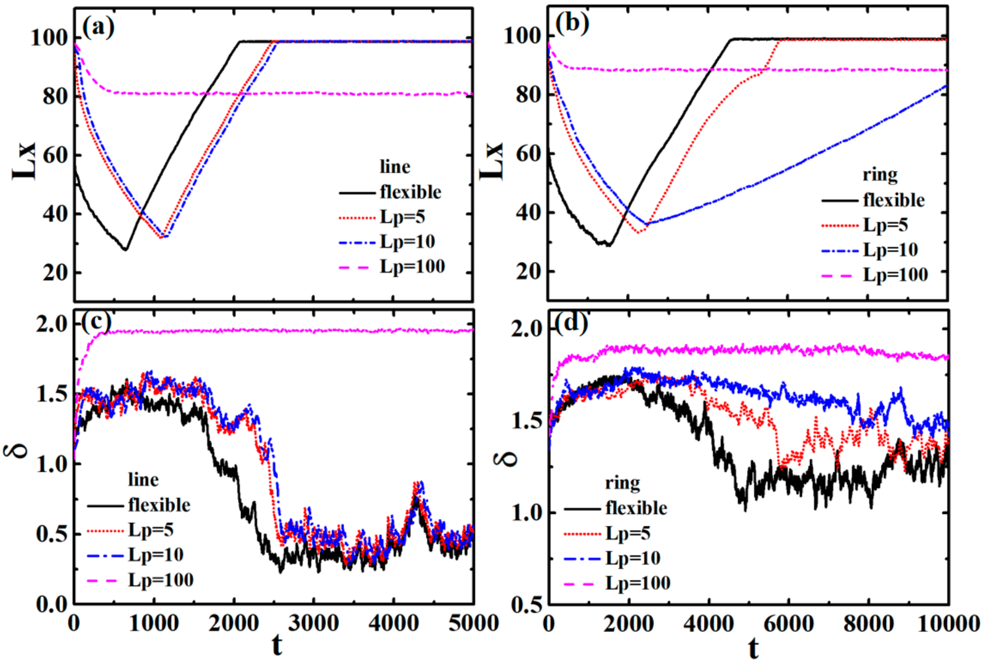

Figure S3. The length along the tube Lx and the thickness δ as a function of time t for linear and ring polymers with different rigidities under the external force f=1.0.

Figure S4 shows the compression processes of polymer chains with various rigidities under different magnitude of external force. The flexible linear polymer is firstly compressed and then stretched, as shown in Figure S4(a) and Figure S4(d). As shown in Figure S4(b) and Figure S4(e), the semiflexible polymers only present slight compression under the weak force (f=0.1). They also are compressed and then stretched at larger external force. For rigid chains, only the large force can overwhelm their bending energy (f=10.0) and polymers are compressed into the collapsed states, as shown in Figure S4(c) and Figure S4(f).

We also choose the chain states with the minimum size along the direction of external force as the finally collapsed states.

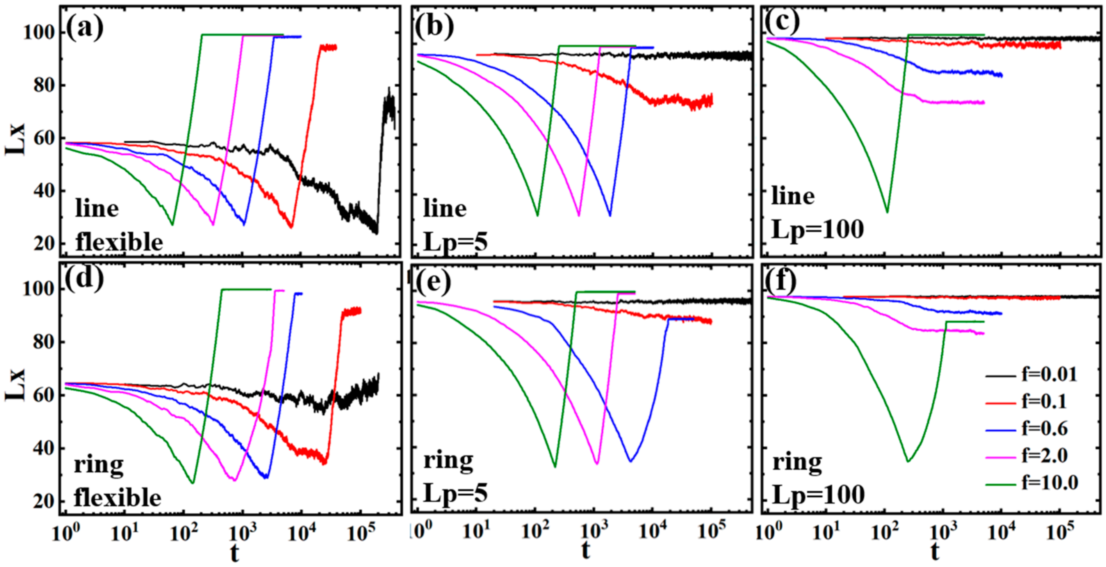

Figure S4. The trajectory of the length along the tube Lx for linear and ring polymers under different magnitude of external force.
